# The Association between Coffee Consumption Pattern and Prevalence of Metabolic Syndrome in Korean Adults

**DOI:** 10.3390/nu11122992

**Published:** 2019-12-06

**Authors:** Seong-Ah Kim, Sangah Shin

**Affiliations:** Department of Food and Nutrition, Chung-Ang University, Gyeonggi-do 17546, Korea; sakim8864@gmail.com

**Keywords:** 3-in-1 coffee, black coffee, coffee, metabolic syndrome

## Abstract

The inconsistent results of epidemiologic studies suggest that the health effects of coffee vary depending on coffee consumption pattern, such as the type and amount of coffee intake. This study investigated the association between coffee consumption and metabolic syndrome (MetS) in Korean adults. In total, coffee consumption patterns in 14,132 participants were assessed based on two-day, 24-h recall data. Multivariable logistic regression was used to examine the association between the type and daily servings of coffee and the prevalence of MetS. In women, the prevalence of MetS (odds ratio (OR) 0.82; 95% confidence interval (CI): 0.70, 0.96), elevated triglycerides (0.85; 0.75, 0.97), and reduced high-density lipoprotein (HDL)-cholesterol (HDL-C; 0.74; 0.66, 0.83) in **3**-in-1 coffee consumers, as well as the prevalence of increased waist circumference (0.81; 0.68, 0.98) and reduced HDL-C (0.68; 0.59, 0.80) in black coffee consumers, were significantly lower compared to non-coffee consumers. Also, the inverse associations between total coffee intake, black coffee intake, and **3**-in-1 coffee intake with MetS or components of MetS were more significant in individuals who consumed >1 versus ≤1 serving/day. In conclusion, coffee consumption (regardless of type) was associated with a reduced prevalence of MetS and its components in Korean women.

## 1. Introduction

Coffee, which has a distinctive taste and smell, is one of the world’s most popular drinks. In recent decades, far-reaching developments in the food industry and rapid changes in dietary lifestyles have led to increased coffee consumption in Koreans [1]. In particular, there is a unique type of coffee in Korea called “**3**-in-1 coffee” that consists of a small sachet containing instant coffee, nondairy creamer, and sugar [1]. This instant coffee accounts for a significant portion of coffee consumption in Korean adults [2], suggesting a diversity of coffee consumption patterns.

Coffee contains both caffeine [3] and polyphenol, which has antioxidant properties [4,5]. Epidemiologic studies indicate that higher coffee consumption is associated with lower risks of type 2 diabetes [6,7], increased blood pressure [8], cardiovascular disease, and all-cause mortality [9,10]. However, other studies reported that certain types of coffee increase all-cause mortality [11] and that coronary heart disease might be increased because coffee raises cholesterol concentrations [12]. Also, the intake of coffee with additives such as sugar or creamer was associated with increased obesity, abdominal obesity [13], and metabolic syndrome (MetS) in Koreans [2,14].

MetS is a group of metabolic abnormalities including atherogenic dyslipidemia, elevated blood pressure, and plasma glucose [15]. Insulin resistance is a hallmark feature of MetS and key risk factor for development of type 2 diabetes and cardiovascular diseases [16]. Coffee consumption has been reported to prevent the insulin resistance by altering physiological signals related to food intake and intestinal hormone secretion [17]. In particular, polyphenols in coffee such as chlorogenic acids (CGAs) reduce intestinal glucose absorption, resulting in reduced glucose-dependent insulinotropic polypeptide (GIP) and increased glucagon-like peptide 1 (GLP-1) levels, which can improve insulin sensitivity [17]. However, there have been conflicting evidence supporting that caffeine in coffee mediates its negative effects on glucose tolerance via adenosine receptor antagonism and epinephrine secretion [18,19] Also, coffee consumption was associated with circulating levels of leptin and ghrelin, which are peptides that regulate appetite, satiety, and energy metabolism [20,21]. Thus, coffee consumption may affect the major endocrine system, which influences energy homeostasis, glucose, and lipid metabolism.

The inconsistent results suggest that the health effects of coffee may vary depending on the coffee consumption pattern, such as coffee type and amount of intake. However, there is a lack of research considering both qualitative and quantitative information about coffee consumption patterns. Therefore, this study examined the typical coffee consumption patterns of Korean adults based on 24-h recall data and investigated the association between coffee consumption patterns and MetS.

## 2. Materials and Methods

### 2.1. Study Population

This study was cross-sectional and based on a large-scale, community-based genomic cohort known as the Health Examinees (HEXA) study. In total, 167,169 participants aged 40–69 years were recruited for the HEXA study between 2004 and 2013 [22]. A subset of the participants (*n* = 33,607) completed the 24-h recall. Among these, participants who completed two-day, 24-h recall (*n* = 18,934) were included in the present study. Individuals with insufficient data for a diagnosis of MetS, such as blood lipid profile or blood glucose level (*n* = 42) and those with low or high energy intake (<800 or ≥4000 kcal/day in men or <500 or ≥3500 kcal/day in women; *n* = 48), were excluded. Furthermore, we excluded people with history of stroke (*n* = 217) or ischemic heart diseases (*n* = 533), and those who take drugs to treat hypertension, diabetes, and hyperlipidemia (*n* = 3962). Finally, 14,132 participants (3862 men and 10,270 women) were included in this study. The HEXA study was approved by the Ethics Committee of the Korean Health and the institutional review boards of all participating hospitals (IRB No. E-1503-103-657). All individuals provided written informed consent before participation.

### 2.2. Assessment of Coffee Consumption

Two-day, 24-h recall data were used to assess the participants’ coffee consumption. Black coffee was defined as coffee powder or extracts, potentially with added water but no other ingredients. “**3**-in-1 coffee” was defined as instant coffee drinks containing coffee powder, creamer, and sugar. Other coffee referred to other types of coffee, excluding black coffee and **3**-in-1 coffee. Participants were classified according to the type of coffee they consumed: Non-coffee consumer, black coffee consumer, **3**-in-1 coffee consumer, other coffee or combined coffee consumer, and coffee consumer. If any coffee type appeared at least once in a person’s two-day, 24-h recall, the individual was classified as a coffee consumer. Individuals who consumed only black coffee or **3**-in-1 coffee were classified as black-coffee consumers or **3**-in-1 coffee consumers, respectively. Individuals who consumed other coffee or several types of coffee were classified as other coffee or combined coffee consumers.

### 2.3. Anthropometric Measurement and Diagnosis of MetS

Body mass index (BMI) values were used to classify participants as follows: Underweight (<18.5 kg/m^2^), normal (≥18.5 and <23 kg/m^2^), overweight (≥23 and <25 kg/m^2^), and obese (≥25 kg/m^2^), according to the International Obesity Task Force for adults in Asian and Pacific regions [23]. MetS was defined according to criteria of the updated National Cholesterol Education Program Adult Treatment Panel III (NCEP ATP III) [24], with a modified waist circumference (WC) cutoff for Asians. Participants who satisfied at least three of the following five criteria were diagnosed with MetS: WC ≥ 90 cm in men or ≥ 80 cm in women; blood triglyceride (TG) level ≥150 mg/dL; blood high-density lipoprotein cholesterol (HDL-C) level <40 mg/dL in men or <50 mg/dL in women; systolic blood pressure (BP) ≥130 mmHg or diastolic BP ≥85 mmHg; and fasting blood glucose (FBG) level ≥100 mg/dL.

### 2.4. Assessment of Other Variables

Data about sociodemographic variables, such as age, sex, and education level, and lifestyle variables, such as alcohol consumption, current smoking status, and physical activity, were obtained through self-administered questionnaires. Education level was divided into three categories: Middle school or under, high school, and college or above. Alcohol consumption was divided into four categories according to the estimated daily alcohol intakes: Non-drinker, <25 g/day, 25–49 g/day, ≥50 g/day. Current smoking status was divided into three categories: Current smoker, past smoker, or never smoker. Physical activity was divided into two categories: Active (performed physical activity for ≥30 min once a day for ≥5 days per week) or inactive.

### 2.5. Statistical Analysis

Participants’ general characteristics, such as age, education level, BMI, body weight status, alcohol consumption, current smoking status, physical activity, nutrient intake, and MetS parameters according to coffee consumption patterns by sex, were compared using a generalized linear model (GLM) for continuous variables and a chi-square test for categoric variables.

A multivariable logistic regression model was used to examine the association between coffee consumption and MetS and its components. The 95% confidence intervals (CIs) of odds ratios (ORs) according to coffee type and daily servings of coffee were estimated after adjusting for age (continuous), BMI, energy intake (continuous), education level, alcohol consumption, smoking status, and physical activity. Linear trends across increasing categories of coffee consumption were tested by assigning the median of each category as a continuous variable. Statistical significance was defined as a two-sided *p* value <0.05. All analyses were performed using SAS software (version 9.4; SAS Institute Inc., Cary, NC, USA).

## 3. Results

Table 1 shows the study population’s general characteristics according to coffee consumption pattern by sex. In both men and women, the proportion of participants with college or above education was greatest for black coffee consumers (*p* < 0.0001) and the average daily energy intake was highest for **3**-in-1 coffee consumers (*p* < 0.0001). In men, mean BMI, WC, blood TG, and FBG levels were significantly higher in black coffee consumers than in other groups (all *p* < 0.05). In women, mean BMI and WC were significantly higher in **3**-in-1 coffee consumers than in other groups (all *p* < 0.05).

Table 2 presents the multivariable-adjusted OR and 95% CI for MetS, including its components, according to the type of coffee consumed by sex. In men, there was no significant association between coffee type and MetS. In women, the prevalence of MetS (OR 0.82; 95% CI: 0.70, 0.96), elevated TG (OR 0.85; 95% CI: 0.75, 0.97), and reduced HDL-C (OR 0.74; 95% CI: 0.66, 0.83) was significantly lower in **3**-in-1 coffee consumers than non-coffee consumers. In black-coffee consumers, the prevalence of increased WC (OR 0.81; 95% CI: 0.68, 0.98), and reduced HDL-C (OR 0.68; 95% CI: 0.59, 0.80) was significantly lower than in non-coffee consumers. Regardless of the coffee type, coffee consumers versus non-coffee consumers had lower ORs of the following: Increased WC (−12%; OR 0.88; 95% CI: 0.77, 0.99), elevated TG (−13%; OR 0.87; 95% CI: 0.77, 0.98), and reduced HDL-C (−27%; OR 0.73; 95% CI: 0.66, 0.81).

Table 3 shows the multivariable-adjusted OR and 95% CI for MetS according to daily servings of coffee by sex. In men, individuals who consumed >1 serving/day of coffee had a 19% lower prevalence of elevated BP (OR 0.81; 95% CI: 0.67, 0.97; *p* for trend = 0.0101) compared to non-coffee consumers. Also, men who consumed >1 serving/day of **3**-in-1 coffee had a significantly lower prevalence of reduced HDL-C (OR 0.72; 95% CI: 0.56, 0.94; *p* for trend = 0.0314) and elevated BP (OR 0.76; 95% CI: 0.62, 0.93; *p* for trend = 0.0071) compared to non-coffee consumers. In women, the prevalence of MetS (>1 serving/day: OR 0.84; 95% CI: 0.71, 0.99; *p* for trend = 0.0436) and some components, such as elevated TG (>1 serving/day: OR 0.79; 95% CI: 0.68, 0.91; *p* for trend = 0.0007) and reduced HDL-C (>1 serving/day: OR 0.65; 95% CI: 0.57, 0.73; *p* for trend < 0.0001), significantly decreased as total coffee intake increased. Women who consumed >1 serving/day of black coffee had a significantly lower prevalence of MetS (OR 0.63; 95% CI: 0.45, 0.88; *p* for trend = 0.0089), increased WC (OR 0.71; 95% CI: 0.54, 0.93; *p* for trend = 0.0138) and reduced HDL-C (OR 0.47; 95% CI: 0.36, 0.61; *p* for trend < 0.0001) compared to non-coffee consumers and those who consumed ≤1 serving/day. Women who consumed >1 serving/day of **3**-in-1 coffee had a significantly lower prevalence of elevated TG (OR 0.73; 95% CI: 0.60, 0.88; *p* for trend = 0.0010) and reduced HDL-C (OR 0.67; 95% CI: 0.57, 0.79; *p* for trend < 0.0010) compared to non-coffee consumers and those who consumed ≤1 serving/day.

## 4. Discussion

In the present study, which examined the association between coffee consumption pattern and MetS in the Korean population on a large scale, we found that both **3**-in-1 coffee and black coffee had inverse associations with MetS and its components. Also, these inverse associations between the intake of total, black, or **3**-in-1 coffee with MetS and its components were more significant in individuals who consumed >1 versus ≤1 serving/day.

The consumption of total and black coffee was significantly associated with a lower prevalence of abdominal obesity. These observations are inconsistent with those of previous observational studies. In a cross-sectional study in the United States, the frequency of coffee consumption was not associated with abdominal obesity [25], while coffee consumption was positively associated with a higher prevalence of abdominal obesity in the Republic of Korea [1,2]. This inconsistency may be attributed to differences in the study population and diet assessment method. The previous studies used a food frequency questionnaire (FFQ) to assess coffee consumption, which showed recall bias, leading to underestimation of true coffee consumption [26]. Moreover, the use of FFQ with closed-ended response categories causes nondifferential misclassification that could bias study results [27]. In the present study, we used two-day, 24-h recall to estimate participants’ usual coffee consumption, thus avoiding the effects of these biases.

We found that women who consumed >1 serving/day of coffee or **3**-in-1 coffee were less likely to have increased blood TG levels in women. This finding is consistent with results from previous studies in other countries. In a cross-sectional study in Japan, the United Kingdom, and Norway, moderate coffee consumption was inversely associated with high TG levels [28,29,30]. A positive association observed in women between coffee consumption and blood HDL-C level was also shown in a study conducted in Japan [28], while coffee consumption had no significant association with blood HDL-C level in other European studies [29,30].

In the present study, there was no significant association between coffee consumption pattern and prevalence of elevated FBG, but a significant inverse association between coffee consumption and prevalence of elevated TG was observed. The underlying mechanism for the TG-lowering effect of coffee consumption can be explained by the role of CGAs on limiting glucose absorption from the intestine and reducing insulin secretion via lowered glucose-dependent GIP response [17]. Reduced plasma insulin concentrations prevent very low-density lipoprotein (VLDL) synthesis, which can lead to hypertriglyceridemia [31].

We hypothesized that coffee containing additives, such as sugar or creamer, has a less beneficial or unfavorable health effect compared to black coffee. In previous studies in Korea, **3**-in-1 coffee drinkers reported a higher prevalence of MetS and its components compared to non-coffee consumers or black coffee consumers [2,14]. However, the present study showed that **3**-in-1 coffee consumption was similarly beneficial for MetS and its components compared to black coffee. Possible factors contributing to these results are the cross-sectional design, the potential effect of coffee creamer on blood HDL-C, and that using limited amounts of additives does not affect health. First, though we excluded the patients taking drugs to treat hypertension, diabetes, and hyperlipidemia in this study, it is possible that participants with poor metabolic health refrained from consuming **3**-in-1 coffee for health reasons at the time of the survey. This intentional diet change in participants may confound the results, weakening the potential negative effects of **3**-in-1 coffee on the risk of MetS. Second, coffee creamer, which is high in fat, may increase blood HDL-C levels and play a counterproductive role against reduced HDL-C. Third, the added sugar might not be enough to increase blood glucose or TG levels. In fact, one serving of **3**-in-1 coffee includes 5.5 g of sugar, which contains approximately 21.4 kcal. According to Korean Dietary Recommended Intakes, sugars added during cooking and processing should be within 10% of the total energy intake [32]. Assuming 1,900 kcal as an estimated daily energy requirement for women in their 40s, the amount of added sugar in one serving of **3**-in-1 coffee is approximately 1.1%. Thus, one or two servings of **3**-in-1 coffee per day can be expected to have no adverse health effects.

In the present study, women who consumed >1 serving/day of black coffee, **3**-in-1 coffee, or total coffee had a significantly lower prevalence of MetS or its components compared to non-coffee consumers and those who consumed ≤1 serving/day. However, these results do not imply that high coffee consumption should be encouraged. Excessive coffee intake is undesirable because high caffeine intake has adverse effects, such as general toxicity, cardiovascular effects, and effects on bone status and calcium balance in healthy adults [33]. According to a review study, moderate daily caffeine intake (≤400 mg/day) is not associated with adverse effects [33]. Instead, moderate amounts of caffeine increase energy availability and daily energy expenditure and decrease fatigue [34]. Therefore, an appropriate level of coffee consumption should be recommended to minimize the adverse effects of caffeine and maximize its beneficial effects. However, heavy caffeine intake during pregnancy has been associated with a risk of fetal growth retardation, resulting in low birth weight [35,36]. Consequently, pregnant women in the at-risk population may require specific advice about limiting caffeine intake.

In the present study, coffee consumption patterns differed by sex and age group. The proportion of non-coffee consumers (25.9%) in women was higher than in men (22.4%). Women have a higher proportion of black coffee consumers (14.1%) than men (8.6%), but a lower proportion of **3**-in-1 coffee consumers (37.3%) than men (51.9%). As age increased, the proportion of people who did not consume coffee increased, showing that the proportion of non-coffee consumers in women ≥ 60 years (39.6%) was more than twice compared to women in their 40s (19.6%).

Sex differences were associated with coffee consumption and MetS in our study. No significant association between coffee type with MetS and its components was observed in men, while MetS and its components were significantly associated with different types of coffee and total coffee in women. These differences might result from differences in lifestyle factors between men and women. For example, lifestyle factors such as alcohol consumption and smoking status were less heterogeneous in women (mostly non-smokers and non-drinkers). Thus, there was a possibility that the effects of dietary factors might have been more significant in women than men.

In the Republic of Korea, the prevalence of MetS increased significantly from 24.9% in 1998 to 31.3% in 2007 [37]. Among the five components of MetS, the occurrence of low HDL-C increased the most over 10 years, by 13.8%, followed by abdominal obesity (8.7%) and hypertriglyceridemia (4.9%) [37]. The results of our study showed that black coffee, **3**-in-1 coffee, and total coffee consumption were inversely associated with MetS. All types of coffee consumption were inversely associated with reduced HDL-C, and black coffee and total coffee consumption were associated with a lower prevalence of abdominal obesity. These results suggest that, regardless of type, coffee might play a preventive role in the development of MetS. However, further prospective studies or well-designed randomized controlled trials should be conducted to demonstrate the preventive effects of coffee intake on MetS and its components.

This study has several limitations. First, its cross-sectional nature means that causal associations could not be confirmed. Second, we did not include the amounts of caffeine, added sugar, and creamer in the statistical model. Last, there was potential for unmeasured and residual confounding factors, which are a common problem in observational studies.

Despite these limitations, our study has several strengths. First, because we estimated participants’ coffee consumption patterns based on two-day, 24-h recall data, it was possible to obtain relatively accurate information about usual coffee intake. Second, we assessed both coffee type and the individual intake of each coffee type. Thus, we can provide both qualitative and quantitative information about coffee consumption. Further investigations will be needed to confirm the causal association between coffee consumption and MetS or related diseases.

## 5. Conclusions

Coffee consumption, regardless of type, was associated with a lower prevalence of MetS and its components in Korean women. Further research is needed to confirm the causal association between coffee consumption and MetS or other related chronic diseases in a prospective study or well-designed randomized controlled trials.

## Figures and Tables

**Table 1:** General characteristics of the study population according to coffee consumption pattern by sex.

Mean ± Standard Deviation	Men (*n* = 3862)					Women (*n* = 10,270)				
	Non-Coffee Consumer (*n* = 865)	Black-Coffee Consumer (*n* = 333)	3-in-1 Coffee Consumer (*n* = 2004)	Other Coffee or Combined Coffee Consumer (*n* = 660)	*p* Value ^1^	Non-Coffee Consumer (*n* = 2661)	Black-Coffee Consumer (*n* = 1451)	3-in-1 Coffee Consumer (*n* = 3827)	Other Coffee or Combined Coffee Consumer (*n* = 2331)	*p* Value ^1^
Age (year)	54.5 ± 8.7	52.0 ± 8.2	52.9 ± 8.3	52.2 ± 8.0	<0.0001	53.4 ± 7.8	49.9 ± 6.9	51.1 ± 7.5	50.1 ± 7.3	<0.0001
Age (year), *n* (%)										
40–49	266 (30.8)	139 (41.7)	719 (35.9)	259 (39.2)	<0.0001	836 (31.4)	678 (46.7)	1637 (42.8)	1114 (47.8)	<0.0001
50–59	318 (36.8)	126 (37.8)	796 (39.7)	265 (40.2)		1183 (44.5)	650 (44.8)	1623 (42.4)	927 (39.8)	
≥60	281 (32.5)	68 (20.4)	489 (24.4)	136 (20.6)		642 (24.1)	123 (8.5)	567 (14.8)	290 (12.4)	
Education level, *n* (%)										
Under middle school	184 (21.3)	19 (5.7)	405 (20.2)	96 (14.6)	<0.0001	902 (33.9)	245 (16.9)	1032 (27.0)	513 (22.0)	<0.0001
High school	343 (39.7)	94 (28.2)	885 (44.2)	231 (35.0)		1235 (46.4)	706 (48.7)	1949 (50.9)	1141 (49.0)	
Over college	338 (39.1)	219 (65.8)	707 (35.3)	332 (50.3)		502 (18.9)	493 (34.0)	809 (21.1)	657 (28.2)	
BMI (kg/m^2^) ^2^	23.6 ± 2.7	24.8 ± 2.7	24.1 ± 2.7	24.4 ± 2.7	<0.0001	22.9 ± 2.8	23.2 ± 2.9	23.3 ± 2.9	23.3 ± 2.9	<0.0001
Body weight status, *n* (%)										
Underweight	18 (2.1)	2 (0.6)	37 (1.9)	5 (0.8)	<0.0001	105 (4.0)	38 (2.6)	102 (2.7)	62 (2.7)	<0.0001
Normal	342 (39.5)	85 (25.5)	629 (31.4)	184 (27.9)		1354 (50.9)	712 (49.1)	1836 (48.0)	1073 (46.0)	
Overweight	240 (27.8)	90 (27.0)	619 (30.9)	226 (34.2)		672 (25.3)	360 (24.8)	970 (25.4)	625 (26.8)	
Obese	265 (30.6)	156 (46.9)	719 (35.9)	245 (37.1)		530 (19.9)	341 (23.5)	919 (24.0)	571 (24.5)	
Alcohol consumption, *n* (%)										
Non-drinker	212 (24.5)	68 (20.4))	424 (21.2)	138 (20.9)	0.0052	2079 (78.1)	833 (57.4)	2420 (63.2)	1356 (58.2)	<0.0001
<25 g/day	403 (46.6)	178 (53.5)	1043 (52.1)	344 (52.1)		503 (18.9)	562 (38.7)	1304 (34.1)	893 (38.3)	
25-49 g/day	87 (10.1)	44 (13.2)	260 (13.0)	94 (14.2)		23 (0.9)	20 (1.4)	37 (1.0)	29 (1.2)	
≥50 g/day	98 (11.3)	27 (8.1)	172 (8.6)	51 (7.7)		9 (0.3)	8 (0.6)	17 (0.4)	9 (0.4)	
Current smoking status, *n* (%)										
Never smoked	311 (36.0)	98 (29.4)	457 (22.8)	149 (22.6)	<0.0001	2599 (97.7)	1379 (95.0)	3680 (96.2)	2230 (95.7)	0.0001
Past smoker	383 (44.3)	165 (49.6)	765 (38.2)	287 (43.5)		28 (1.1)	27 (1.9)	40 (1.1)	29 (1.2)	
Current smoker	171 (19.8)	69 (20.7)	780 (38.9)	223 (33.8)		32 (1.2)	45 (3.1)	106 (2.8)	70 (3.0)	
Physical activity ^3^, *n* (%)										
Active	200 (23.1)	75 (22.5)	322 (16.1)	130 (19.7)	0.0002	590 (22.2)	318 (21.9)	654 (17.1)	465 (20.0)	<0.0001
Inactive	665 (76.9)	257 (77.2)	1680 (83.8)	529 (80.2)		2069 (77.8)	1133 (78.1)	3170 (82.8)	1864 (80.0)	
Nutrient intake										
Total energy (kcal/day)	1568.7 ± 334.5	1609.4 ± 332.1	1649.2 ± 327.5	1641.4 ± 323.2	<0.0001	1422.0 ± 331.9	1370.7 ± 332.8	1464.7 ± 326.5	1442.5 ± 337.9	<0.0001
Carbohydrates (% of energy)	66.4 ± 8.5	64.1 ± 8.9	65.1 ± 8.1	64.7 ± 8.5	<0.0001	68.6 ± 7.5	65.7 ± 8.1	66.9 ± 7.5	66.2 ± 7.7	<0.0001
Protein (% of energy)	15.8 ± 2.7	15.8 ± 2.4	15.5 ± 2.5	15.7 ± 2.5	0.0193	15.4 ± 2.6	15.8 ± 2.7	15.2 ± 2.5	15.5 ± 2.6	<0.0001
Fat (% of energy)	18.1 ± 5.8	19.8 ± 6.0	19.1 ± 5.6	19.5 ± 5.9	<0.0001	18.2 ± 5.8	20.4 ± 6.3	19.6 ± 5.8	20.0 ± 5.9	<0.0001
Metabolic syndrome parameter										
Waist circumference (cm)	83.6 ± 7.7	85.8 ± 7.3	84.5 ± 7.3	85.1 ± 6.9	<0.0001	76.8 ± 7.7	76.8 ± 7.7	77.3 ± 7.8	77.1 ± 7.9	0.0362
Blood triglyceride (mg/dL)	141.0 ± 108.1	158.6 ± 106.0	150.2 ± 105.5	146.8 ± 88.6	0.0375	114.0 ± 75.8	111.2 ± 82.5	107.4 ± 64.6	107.5 ± 65.9	0.0008
Blood HDL cholesterol (mg/dL)	51.7 ± 13.5	50.1 ± 12.4	50.3 ± 12.1	49.7 ± 11.8	0.0069	56.8 ± 13.4	60.1 ± 14.0	58.6 ± 13.5	58.9 ± 13.3	<0.0001
Fasting blood glucose (mg/dL)	95.8 ± 13.6	97.8 ± 19.5	96.0 ± 14.3	97.4 ± 19.8	0.0546	92.3 ± 12.7	91.8 ± 12.6	91.6 ± 11.6	92.1 ± 12.6	0.1467
Systolic blood pressure (mmHg)	123.6 ± 14.8	124.1 ± 14.8	123.8 ± 14.0	124.2 ± 13.9	0.8521	119.5 ± 14.5	118.7 ± 15.1	119.1 ± 14.8	118.9 ± 14.6	0.2761
Diastolic blood pressure (mmHg)	76.8 ± 10.1	77.7 ± 10.2	77.1 ± 9.7	77.1 ± 9.2	0.5981	73.1 ± 9.3	73.0 ± 9.8	73.1 ± 9.4	73.0 ± 9.5	0.9898

BMI, body mass index. ^1^ *p* values were calculated by generalized linear model for continuous variables and χ^2^ test for categorical variables. ^2^ Underweight (BMI < 18.5 kg/m^2^); normal (BMI ≥18.5 and <23 kg/m^2^); overweight (BMI ≥23 and <25 kg/m^2^); obese (BMI ≥ 25 kg/m^2^). ^3^ Active (performed physical activity for ≥30 min once a day for ≥5 days per week).

**Table 2:** Multivariable-adjusted odds ratios and 95% CIs for metabolic syndrome (including its components) according to type of coffee consumed (by sex).

	Non-Consumer (Reference)	Black-Coffee Consumer	3-in-1 Coffee Consumer	Other Coffee or Combined Coffee Consumer	Coffee Consumer
Men					
MetS					
Model 1	Ref	1.20 (0.85–1.69)	0.88 (0.70–1.11)	0.97 (0.73–1.29)	0.93 (0.75–1.16)
Model 2	0.84 (0.59–1.18)	Ref	0.73 (0.54–1.00)	0.81 (0.57–1.14)	-
Increased WC					
Model 1	Ref	0.87 (0.59–1.27)	0.80 (0.62–1.03)	0.83 (0.61–1.14)	0.82 (0.64–1.04)
Model 2	1.15 (0.79–1.69)	Ref	0.92 (0.65–1.31)	0.96 (0.65–1.41)	-
Elevated TG					
Model 1	Ref	1.13 (0.85–1.49)	1.02 (0.85–1.23)	1.06 (0.85–1.33)	1.04 (0.88–1.24)
Model 2	0.89 (0.67–1.18)	Ref	0.91 (0.70–1.18)	0.94 (0.71–1.26)	-
Reduced HDL-C					
Model 1	Ref	0.99 (0.70–1.38)	0.78 (0.62–0.97)	1.02 (0.78–1.33)	0.85 (0.69–1.05)
Model 2	1.01 (0.72–1.42)	Ref	0.79 (0.58–1.08)	1.03 (0.73–1.45)	-
Elevated BP					
Model 1	Ref	1.04 (0.79–1.36)	0.83 (0.70–0.99)	0.98 (0.79–1.22)	0.88 (0.75–1.04)
Model 2	0.97 (0.74–1.26)	Ref	0.80 (0.62–1.03)	0.95 (0.72–1.25)	-
Elevated FBG					
Model 1	Ref	1.04 (0.78–1.39)	0.93 (0.78–1.12)	0.98 (0.78–1.24)	0.96 (0.80–1.14)
Model 2	0.96 (0.72–1.28)	Ref	0.90 (0.69–1.17)	0.94 (0.70–1.27)	-
Women					
MetS					
Model 1	Ref	0.88 (0.72–1.08)	0.82 (0.70–0.96)	0.97 (0.82–1.16)	0.88 (0.76–1.00)
Model 2	1.13 (0.92–1.39)	Ref	0.93 (0.77–1.13)	1.10 (0.90–1.35)	-
Increased WC					
Model 1	Ref	0.81 (0.68–0.98)	0.91 (0.79–1.05)	0.86 (0.73–1.00)	0.88 (0.77–0.99)
Model 2	1.23 (1.02–1.48)	Ref	1.12 (0.94–1.33)	1.05 (0.88–1.27)	-
Elevated TG					
Model 1	Ref	0.93 (0.78–1.11)	0.85 (0.75–0.97)	0.87 (0.75–1.01)	0.87 (0.77–0.98)
Model 2	1.07 (0.90–1.28)	Ref	0.92 (0.78–1.08)	0.93 (0.78–1.11)	-
Reduced HDL-C					
Model 1	Ref	0.68 (0.59–0.80)	0.74 (0.66–0.83)	0.74 (0.65–0.84)	0.73 (0.66–0.81)
Model 2	1.46 (1.25–1.71)	Ref	1.08 (0.93–1.26)	1.08 (0.92–1.27)	-
Elevated BP					
Model 1	Ref	1.06 (0.91–1.25)	0.98 (0.87–1.11)	1.04 (0.90–1.19)	1.01 (0.91–1.13)
Model 2	0.94 (0.80–1.10)	Ref	0.92 (0.80–1.07)	0.97 (0.83–1.14)	-
Elevated FBG					
Model 1	Ref	1.11 (0.93–1.33)	0.98 (0.85–1.13)	1.14 (0.98–1.34)	1.05 (0.93–1.19)
Model 2	0.90 (0.75–1.08)	Ref	0.88 (0.74–1.05)	1.03 (0.86–1.23)	-

CIs, confidence intervals; BP, blood pressure; FBG, fasting blood glucose; HDL-C, HDL cholesterol; MetS, metabolic syndrome; TG, triglyceride; WC, waist circumference. Model 1: Reference = non-coffee consumer. Adjusted for age (continuous), BMI, energy intake (continuous), education level (under elementary school, middle school, high school, over college), alcohol consumption (non-drinker, <25 g/day, 25−49 g/day, ≥50 g/day), smoking status (never smoker, past smoker, current smoker), and physical activity (active or inactive). Model 2: Reference = black-coffee consumer. Adjusted for age (continuous), BMI, energy intake (continuous), education level (under elementary school, middle school, high school, over college), alcohol consumption (non-drinker, <25 g/day, 25−49 g/day, ≥50 g/day), smoking status (never smoker, past smoker, current smoker), and physical activity (active or inactive).

**Table 3:** Multivariable-adjusted odds ratios and 95% CIs for metabolic syndrome (including its components) according to daily servings of coffee (by sex).

		Total Coffee			Black Coffee			3-in-1 Coffee		
	Non-Coffee Consumer (reference)	≤1 Serving/Day	>1 Serving/Day	*p* for Trend ^1^	≤1 Serving/Day	>1 Serving/Day	*p* for Trend ^1^	≤1 Serving/Day	>1 Serving/Day	*p* for Trend ^1^
Men										
*n*	865	1378	1619		184	149		983	1021	
MetS	1.00	0.99 (0.78–1.27)	0.88 (0.69–1.12)	0.2242	1.18 (0.77–1.81)	1.26 (0.77–2.05)	0.3442	0.94 (0.73–1.23)	0.85 (0.65–1.11)	0.2274
Increased WC	1.00	0.78 (0.60–1.02)	0.85 (0.66–1.11)	0.3900	0.69 (0.42–1.14)	1.14 (0.66–1.94)	0.6910	0.73 (0.55–0.98)	0.88 (0.66–1.18)	0.9306
Elevated TG	1.00	1.12 (0.92–1.35)	0.98 (0.81–1.18)	0.5701	1.17 (0.82–1.69)	1.23 (0.82–1.83)	0.3024	1.13 (0.92–1.39)	0.94 (0.76–1.16)	0.2464
Reduced HDL-C	1.00	0.90 (0.71–1.13)	0.81 (0.64–1.02)	0.0680	1.09 (0.71–1.66)	0.93 (0.57–1.51)	0.7890	0.80 (0.63–1.03)	0.72 (0.56–0.94)	0.0314
Elevated BP	1.00	0.97 (0.81–1.16)	0.81 (0.67–0.97)	0.0101	1.17 (0.83–1.64)	0.95 (0.65–1.40)	0.8577	0.90 (0.74–1.10)	0.76 (0.62–0.93)	0.0071
Elevated FBG	1.00	0.98 (0.81–1.19)	0.94 (0.77–1.14)	0.4749	0.92 (0.64–1.34)	1.13 (0.75–1.69)	0.5978	0.96 (0.78–1.18)	0.92 (0.75–1.15)	0.5016
Women										
*n*	2661	4377	3232		909	542		2515	1312	
MetS	1.00	0.90 (0.78–1.04)	0.84 (0.71–0.99)	0.0436	0.97 (0.77–1.23)	0.63 (0.45–0.88)	0.0089	0.82 (0.69–0.97)	0.82 (0.66–1.02)	0.1032
Increased WC	1.00	0.88 (0.77–1.01)	0.87 (0.75–1.01)	0.0989	0.90 (0.72–1.11)	0.71 (0.54–0.93)	0.0138	0.88 (0.75–1.02)	0.99 (0.82–1.20)	0.9110
Elevated TG	1.00	0.92 (0.81–1.05)	0.79 (0.68–0.91)	0.0007	0.99 (0.81–1.21)	0.81 (0.61–1.06)	0.1308	0.90 (0.78–1.04)	0.73 (0.60–0.88)	0.0010
Reduced HDL-C	1.00	0.79 (0.70–0.88)	0.65 (0.57–0.73)	<0.0001	0.82 (0.69–0.98)	0.47 (0.36–0.61)	<0.0001	0.77 (0.68–0.88)	0.67 (0.57–0.79)	<0.0001
Elevated BP	1.00	1.03 (0.92–1.16)	0.98 (0.86–1.11)	0.5853	1.03 (0.86–1.24)	1.02 (0.80–1.30)	0.8473	1.02 (0.89–1.16)	0.92 (0.78–1.08)	0.2806
Elevated FBG	1.00	1.05 (0.92–1.21)	1.05 (0.90–1.22)	0.6429	1.15 (0.93–1.42)	0.99 (0.75–1.30)	0.9876	0.98 (0.84–1.15)	0.93 (0.77–1.13)	0.4732

CIs, confidence intervals; BP, blood pressure; FBG, fasting blood glucose; HDL-C, HDL cholesterol; MetS, metabolic syndrome; TG, triglyceride; WC, waist circumference. ^1^ *p* for trend values across increasing categories of coffee consumption were tested by assigning the median of each category and as a continuous variable in the logistic regression model after adjusting for age (continuous), BMI, energy intake (continuous), education level (under elementary school, middle school, high school, over college), alcohol consumption (non-drinker, <25 g/day, 25–49 g/day, ≥50 g/day), smoking status (never smoker, past smoker, current smoker), and physical activity (active or inactive).

## References

[1] Je Y., Jeong S., Park T. (2014). Coffee consumption patterns in Korean adults: The Korean National Health and Nutrition Examination Survey (2001–2011). Asia Pac. J. Clin. Nutr..

[2] Kim H.J., Cho S., Jacobs D.R., Park K. (2014). Instant coffee consumption may be associated with higher risk of metabolic syndrome in Korean adults. Diabetes Res. Clin. Pr..

[3] Barone J.J., Roberts H.R. (1996). Caffeine consumption. Food Chem. Toxicol..

[4] Daglia M., Papetti A., Gregotti C., Bertè F., Gazzani G. (2000). In vitro antioxidant and ex vivo protective activities of green and roasted coffee. J. Agric. Food Chem..

[5] Borrelli R.C., Visconti A., Mennella C., Anese M., Fogliano V. (2002). Chemical characterization and antioxidant properties of coffee melanoidins. J. Agric. Food Chem..

[6] Van Dam R.M., Feskens E.J. (2002). Coffee consumption and risk of type 2 diabetes mellitus. Lancet.

[7] Salazar-Martinez E., Willett W.C., Ascherio A., Manson J.E., Leitzmann M.F., Stampfer M.J., Hu F.B. (2004). Coffee consumption and risk for type 2 diabetes mellitus. Ann. Intern. Med..

[8] Wakabayashi K., Kono S., Shinchi K., Honjo S., Todoroki I., Sakurai Y., Umeda T., Imanishi K., Yoshizawa N. (1998). Habitual coffee consumption and blood pressure: A study of self-defense officials in Japan. Eur. J. Epidemiol..

[9] Crippa A., Discacciati A., Larsson S.C., Wolk A., Orsini N. (2014). Coffee consumption and mortality from all causes, cardiovascular disease, and cancer: A dose-response meta-analysis. Am. J. Epidemiol..

[10] Dawber T.R., Kannel W.B., Gordon T. (1974). Coffee and cardiovascular disease: Observations from the Framingham Study. N. Engl. J. Med..

[11] Sugiyama K., Kuriyama S., Akhter M., Kakizaki M., Nakaya N., Ohmori-Matsuda K., Shimazu T., Nagai M., Sugawara Y., Hozawa A. (2010). Coffee consumption and mortality due to all causes, cardiovascular disease, and cancer in Japanese women. J. Nutr..

[12] Tverdal A., Stensvold I., Solvoll K., Foss O.P., Lund-Larsen P., Bjartveit K. (1990). Coffee consumption and death from coronary heart disease in middle aged Norwegian men and women. BMJ.

[13] Lee J., Kim H., Kim J. (2017). Coffee consumption and the risk of obesity in Korean women. Nutrients.

[14] Yeon J.Y., Bae Y.J. (2017). 3-in-1 coffee consumption is associated with metabolic factors in adults: Based on 2012~2015 Korea National Health and Nutrition Examination Survey. J. Nutr. Health.

[15] Nisoli E., Clementi E., Carruba M.O., Moncada S. (2007). Defective mitochondrial biogenesis: A hallmark of the high cardiovascular risk in the metabolic syndrome?. Circ. Res..

[16] Adiels M., Olofsson S.O., Taskinen M.R., Borén J. (2008). Overproduction of very low–density lipoproteins is the hallmark of the dyslipidemia in the metabolic syndrome. Arterioscler. Thromb. Vasc. Biol..

[17] Tunnicliffe J.M., Shearer J. (2008). Coffee, glucose homeostasis, and insulin resistance: Physiological mechanisms and mediators. Appl. Physiol. Nutr. Metab..

[18] Moisey L.L., Kacker S., Bickerton A.C., Robinson L.E., Graham T.E. (2008). Caffeinated coffee consumption impairs blood glucose homeostasis in response to high and low glycemic index meals in healthy men. Am. J. Clin. Nutr..

[19] Thong F.S., Graham T.E. (2002). Caffeine-induced impairment of glucose tolerance is abolished by β-adrenergic receptor blockade in humans. J. Appl. Physiol..

[20] Zhang Y., Zhang D.Z. (2018). Associations of coffee consumption with circulating level of adiponectin and leptin. A meta-analysis of observational studies. Int. J. Food Sci. Nutr..

[21] Bakuradze T., Parra G.A.M., Riedel A., Somoza V., Lang R., Dieminger N., Hofmann T., Winkler S., Hassmann U., Marko D. (2014). Four-week coffee consumption affects energy intake, satiety regulation, body fat, and protects DNA integrity. Food Res. Int..

[22] Health Examinees Study Group (2015). The Health Examinees (HEXA) study: Rationale, study design and baseline characteristics. Asian Pac. J. Cancer Prev..

[23] International Obesity Task Force & World Health Organization (2000). The Asian-Pacific Perspective: Redefining Obesity and its Treatment.

[24] Grundy S.M., Cleeman J.I., Daniels S.R., Donato K.A., Eckel R.H., Franklin B.A., Gordon D.J., Krauss R.M., Savage P.J., Smith S.C. (2005). Diagnosis and management of the metabolic syndrome—An American Heart Association/National Heart, Lung, and Blood Institute Scientific Statement. Circulation.

[25] Bouchard D.R., Ross R., Janssen I. (2010). Coffee, tea and their additives: Association with BMI and waist circumference. Obes. Facts.

[26] Freedman L.S., Carroll R.J., Wax Y. (1991). Estimating the relation between dietary intake obtained from a food frequency questionnaire and true average intake. Am. J. Epidemiol..

[27] Tylavsky F.A., Sharp G.B. (1995). Misclassification of nutrient and energy intake from use of closed-ended questions in epidemiologic research. Am. J. Epidemiol..

[28] Matsuura H., Mure K., Nishio N., Kitano N., Nagai N., Takeshita T. (2012). Relationship between coffee consumption and prevalence of metabolic syndrome among Japanese civil servants. J. Epidemiol..

[29] Lancaster T., Muir J., Silagy C. (1994). The effects of coffee on serum lipids and blood pressure in a UK population. J. R. Soc. Med..

[30] Stensvold I., Tverdal A., Foss O.P. (1989). The effect of coffee on blood lipids and blood pressure. Results from a Norwegian cross-sectional study, men and women, 40–42 years. J. Clin. Epidemiol..

[31] DeFronzo R.A., Ferrannini E. (1991). Insulin resistance: A multifaceted syndrome responsible for NIDDM, obesity, hypertension, dyslipidemia, and atherosclerotic cardiovascular disease. Diabetes Care.

[32] Ministry of Health and Welfare, The Korean Nutrition Society (2015). Dietary Reference Intakes for Koreans 2015.

[33] Nawrot P., Jordan S., Eastwood J., Rotstein J., Hugenholtz A., Feeley M. (2003). Effects of caffeine on human health. Food Addit. Contam..

[34] Glade M.J. (2010). Caffeine—Not just a stimulant. Nutrition.

[35] Fenster L., Eskenazi B., Windham G.C., Swan S.H. (1991). Caffeine consumption during pregnancy and fetal growth. Am. J. Public Health.

[36] Martin T.R., Bracken M.B. (1987). The association between low birth weight and caffeine consumption during pregnancy. Am. J. Epidemiol..

[37] Lim S., Shin H., Song J.H., Kwak S.H., Kang S.M., Yoon J.W., Choi S.H., Cho S.I., Park K.S., Lee H.K. (2011). Increasing prevalence of metabolic syndrome in Korea: The Korean National Health and Nutrition Examination Survey for 1998–2007. Diabetes Care.

